# A Preclinical Evaluation of *Antrodia camphorata* Alcohol Extracts in the Treatment of Non-Small Cell Lung Cancer Using Non-Invasive Molecular Imaging

**DOI:** 10.1093/ecam/nep228

**Published:** 2011-03-13

**Authors:** Jeng-Feng Chiou, Alexander T. H. Wu, Wei-Tin Wang, Tsu-Hsiang Kuo, Juri G. Gelovani, I-Hsin Lin, Chih-Hsiung Wu, Wen-Ta Chiu, Win-Ping Deng

**Affiliations:** ^1^Cancer Center and Department of Radiatioin Oncology, Taipei Medical University Hospital, Taipei, Taiwan; ^2^Institute of Medical Sciences, National Defence Medical Center, Taipei, Taiwan; ^3^Department of Radiation, School of Medicine, Taipei Medical University, Taipei, Taiwan; ^4^Graduate Institute of Biomedical Materials and Engineering, Taipei Medical University, 250 Wu-Hsing Street, Taipei 110, Taipei, Taiwan; ^5^University of Texas MD Anderson Cancer Center, Houston, TX, USA; ^6^Institute of Traditional Medicine, Chung-Gung University, Taipei, Taiwan; ^7^Department of Surgery, School of Medicine, Taipei Medical University and Hospital, Taipei, Taiwan; ^8^Stem Cell Research Center, Taipei Medical University, Taipei, Taiwan

## Abstract

This study was carried out to provide a platform for the pre-clinical evaluation of anti-cancer properties of a unique CAM (complementary and alternative medicine) agent, *Antrodia camphorata* alcohol extract (ACAE), in a mouse model with the advantageous non-invasive *in vivo* bioluminescence molecular imaging technology. *In vitro* analyses on the proliferation, migration/invasion, cell cycle and apoptosis were performed on ACAE-treated non-small cell lung cancer cells, H441GL and control CGL1 cells. *In vivo*, immune-deficient mice were inoculated subcutaneously with H441GL followed by oral gavages of ACAE. The effect of ACAE on tumor progression was monitored by non-invasive bioluminescence imaging. The proliferation and migration/invasion of H441GL cells were inhibited by ACAE in a dose-dependent manner. In addition, ACAE induced cell cycle arrest at G0/G1 phase and apoptosis in H441GL cells as shown by flow cytometric analysis, Annexin-V immunoflourescence and DNA fragmentation. *In vivo* bioluminescence imaging revealed that tumorigenesis was significantly retarded by oral treatment of ACAE in a dose-dependent fashion. Based on our experimental data, ACAE contains anti-cancer properties and could be considered as a potential CAM agent in future clinical evaluation.

## 1. Introduction

It is now generally accepted that human cancer is the result of a culmination of genetic mutations, which lead to abnormal/pathological signaling pathways contributing to its development and malignancy. For instance, mutations in close to 200 genes have been recently described in a landmark study of human breast and colorectal cancers [1]. This study raises concerns that monofunctional targeted drugs will not cure most common carcinomas. The number of genes that might contribute to carcinogenesis could be much higher as mutations in non-coding regulatory regions of coding genes were not detectable by the methods employed in this study, nor were relative large deletions and insertions, amplifications and translocations. Lung cancer in particular has consistently ranked the leading cause of cancer death for both men and women globally. Two main molecular targets, KRAS and EGFR have been shown to attribute to the development of lung carcinoma and various targeted therapeutic agents have been developed against these two key molecules [2]. However, cancer cells often develop resistance rendering treatments ineffective, indicating the existence of additional signalling pathways and reflecting the complexity of lung cancer etiology. Therefore, the genetic complexity of human cancers warrants the needs to develop new multifunctional drugs to be used either for the prevention or treatment of disease.

Complementary and alternative medicine (CAM) has emerged as an important part of integrative medicine in the management of cancer. Many natural substances have been explored for their unique properties in the treatment of inflammation-related diseases including cancer. *Antrodia camphorata* (AC), a Ganoderma-like fungus of the *Polyporaceae Basidiomycotine* family, has been widely used as a folk medicine in Taiwan for a variety of ailments including abdominal pain, diarrhea, hypertension and suggested to contain anti-inflammatory, anti-oxidative and immune modulating effects [3, 4]. Because wild AC fruiting bodies are very scarce and expensive, artificially cultivated forms have been developed as an alternative. Two major methods for AC cultivation include solid-state culture and liquid-state fermentation. Several bioactive ingredients have been identified in AC including triterpenoid sesquiterpene lactone, steroid and polysaccharides [5–8]. Among these ingredients, triterpenoid has been most studied and demonstrated to exert potent anti-inflammatory and anti-tumorigenic effects both *in vitro* and *in vivo* [9, 10]. Mechanistically, purified or synthetic triterpenoids such as oleanolic acid (OA) and ursolic acid (UA) have been suggested to exert their anti-tumor and cytoprotective effects via a collection of different molecular targets including KEAP1 (the inhibitor of the transcription factor, NRF2), I*κ*B kinase, TGF*β* and STAT signaling pathways. In this regard, extracts obtained from AC represent an ideal new source for the development of multifunctional drug and may act as contributors to the observed anti-cancer properties in a complementary fashion to limit carcinogenesis.

In this study, *Antrodia camphorata* alcohol extracts (ACAE) obtained were examined for their anti-cancer properties in a non-small cell lung cancer (NSCLC) mouse model. ACAE appeared to suppress NSCLC tumorigenesis in a dose-dependent manner. Non-invasive bioluminescence imaging demonstrated oral gavages of ACAE significantly retarded the growth of subcutaneously inoculated NSCLC cells. Mechanistically, we demonstrated that ACAE inhibited NSCLC tumor growth by promoting cell cycle arrest and inducing caspase 3-mediated cellular apoptosis. This study provides rationale and platform for future clinical evaluations of ACAE for the management of NSCL carcinomas.

## 2. Methods

### 2.1. Preparation of ACAE

The artificial culture community of AC fruiting bodies was provided by Well Shine Biotechnology Development Co. (Taipei, Taiwan.) The fine powders of AC fruiting bodies were mixed with 95% ethanol in a 1 : 20 (w/v) ratio and shaken for 24 h at room temperature. The supernatant of extracts was further filtered by a 0.2 mm pore size filter paper (Millex GP Carrigtwohill, Cork, Ireland), centrifuged at 3000 r.p.m. for 30 min to remove the precipitate, and then the extracts were lyophilized and stored at −20°C before use.

### 2.2. Cell Lines

The H441GL cell line was a generous gift from Dr Gelovani's laboratory (University of Texas M.D Anderson Cancer Center, Houston, TX, USA). Briefly, H441 cells originally obtained from the American Type Culture Collection (ATCC) catalogue number: HTB-174. Double reporter genes, enhanced green fluorescent protein (G) and firefly luciferase (L) were integrated permanently into the genome of these cells for non-invasive *in vivo* cell tracking purpose. The control cell line, CGL1 [11, 12] was provided by Dr John-Leslie Redpath from Department of Radiation Oncology, University of California at Irvine, Irvine, CA, USA.

### 2.3. RNA Extraction and Semi-Quantitative PCR Analysis

Total RNA extraction of harvested cells from subconfluent monolayer cultures were extracted using reagent (TRIzol; Invitrogen Life Technologies) and undergone reverse transcription (RT) followed by PCR amplification. Reverse transcription was performed with SuperScript III (Invitrogen Life Technologies) and an Oligo (dT)_12−18_ primer. Four micrograms of RNA were added into a final volume of 21 *μ*L solution containing 10 mM deoxynucleotide triphosphate mix, 10 × RT buffer, 25 mM MgCl_2_, 0.1 M dithiothreitol, RNase inhibitor and RNase H. Four micrograms of RT product were used for the PCR amplification in a final volume of 25 *μ*L containing 2.5 mM deoxynucleotide triphosphate, 25 mM MgCl_2_, upstream or downstream primers, and Taq DNA polymerase (Invitrogen Life Technologies). PCR amplification of reverse-transcribed cDNA was performed with the following primers (forward, F and reverse, R). RB, F: CAT CTA ATG GAC TTC CAG AG; R: CAT AAC AGT CCT AAC TGG AG. p53, F: CAG CCA AGT CTG TGA CTT GCA CGT AC; R: CTA TGT CGA AAA GTG TTT CTG TCA TC. p21, F: ATG TCA GAA CCG GCT GGG GAT G; R: TTA GGG CTT CCT CTT GGA GAA G. Cyclin D1, F: CGA GGA GCT GCT GCA AAT GG; R: GGT ATC AAA ATG CTC CGG AGA GG. Cyclin E, F: ACT GAC TGC TGC TGC CTT GTG C. R: TCG GTG GTG TCA TAA TGC CTC C. Cyclin A, F: GAG CTA TCC TCG TGG ACT GG; R: GCC CAC AAG CTG AAG TTT TC. CXCR4, F: TTC TAC CCC AAT GAC TTG TG; R: ATG TAG TAA GGC AGC CAA CA. Internal control primers used were: GAPDH, F: GCT CTC CAG AAC ATC ATC CCT GCC; R: CGT TGT CAT ACC AGG AAA TGA GCT T. *β*-actin, F: CGG AAC CGC TCA TTG CC; R: ACC ACT GTG CCC ATC TA.

### 2.4. Western Blots

Cell lysates were prepared by treating the cells for 30 min in RIPA lysis buffer (1 × PBS, 1% Nonidet P-40, 0.5% sodium deoxycholate, 0.1% sodium dodecyl sulfate (SDS), protease inhibitor cocktail (Pierce, Rockford, IL, USA). The lysates were centrifuged at 12 000 r.p.m. for 5 min and the protein concentration in the supernatant was determined using a BCA protein assay kit (Pierce, Rockford, IL, USA). Equal amounts of proteins were separated, respectively, by 10% of SDS-polyacrylamide gel electrophoresis and followed by electro-transfer to a PVDF membrane. The membrane was blocked with a solution containing 4% nonfat dried milk TBST buffer (20 mM Tris-HCl pH 7.4, 150 mM NaCl and 0.1% Tween 20) for 1 h and washed with TBST buffer. Protein expression was monitored by immunoblotting using specific primary antibodies and respective secondary antibodies. Caspase 3, cylcins A, D1 and E were purchased from Santa Cruz Biotechnology; cleaved caspase 3 and caspase 9 from Cell Signaling. CXCR4 from AbCam and p53 were from Novocastra, while p21 and RB were from Upstate biotechnology. The incubation conditions were carried out as indicated by the vendor. The signals were visualized using an ECL chemiluminescence kit (Amersham, USA) and UVP imaging system (Upland, CA, USA).

### 2.5. Cell Viability Assay

Inhibition concentration 50% (IC50) for cell viability of ACAE was measured by MTT assay based on the ability of live cells to convert tetrazolium salt into purple formazan. Cells were seeded into 96-well dishes (3 × 10^3^ cells/well) with medium containing ACAE at concentrations ranging from 0 to 300 *μ*g mL^−1^ for 48 h. After treatment for the indicated times, 20 *μ*L of MTT solution (Merck, Darmstadt, German) was added to each well and the plate was incubated at 37°C for 4 h. After the removal of medium, 200 *μ*L of DMSO was added to each well and the plate was gently shaken for 5 min. The absorbance was determined at 540 nm. The cell viability ratio = OD of experimental groups/OD of control groups.

### 2.6. Colony Forming Assay

Both H441GL and CGL1 cells were treated with medium containing ACAE at concentrations ranging from 0 to 300 *μ*g mL for 48 h. Cells were then harvested and seeded in 10 cm culture dish at a density of 500 cell/dish. Fresh culture medium (without ACAE) was replaced on day 7 and approximately on day 10 cell colonies were formed. Cells were then fixed using 4% paraformaldehyde and stained with Crystal violet colony counts.

### 2.7. Cell Scratch Wound Healing Assay

The wound healing assay was performed accordingly. Briefly, when H441GL cells became confluent in a 24-well plate, a wound was made with a sterile tip. Cells were washed with serum-free medium and further incubated in RPMI1640 with 1% FBS with various concentration of ACAE for 12 h. The distance of cell migration at 12 h and 24 h was determined from the photographed images. Wound size% = (wound size at observed time/initial wound size) × 100%.

### 2.8. Migration Assay

A total of 1 × 10^5^ H441GL cells were seeded into the upper Transwell chamber (6.5 mm diameter with a pore size of 8 mm; Corning, Inc.) while in the lower chamber, 800 mL RPMI1640 with 10% FBS was added and the assay was done for 24 h at 37°C and 5% CO_2_. At indicated time points, cells were fixed with 3% formaldehyde and filters were stained with the hematoxylin (Sigma,Inc). Cells that had not migrated from the upper side of the filters were scraped off with a cotton swab, The number of cells that had migrated to the lower side of the filter were counted under a light microscope with five high-power fields (200×). Experiments were done in triplicate.

### 2.9. Gelatin Zymography

Gelatin zymography was performed for both serum free medium control and serum free medium of H441GL with ACAE treatment. SDS-PAGE (7.5% acrylamide and 0.1% gelatin, Sigma-Aldrich) was loaded with 15 *μ*L of total collected culture medium per lane. Electrophoresis was carried out at a constant voltage of 70 V, until the dye reached the bottom of the gel. Following electrophoresis, gels were washed in renaturation buffer (2.5% Triton X-100 in 50 mM Tris-HCl (pH 7.5)) for 1 h with shaking. The zymograms were the incubated for 18 h at 37°C in incubation buffer (0.15 M NaCl, 10 mM CaCl_2_, 0.02% NaN_3_ in 50 mM Tris-HCl (pH 7.5)) Gels were then stained with Coomassie blue and destained with 7% methanol and 5% acetic acid. Areas of enzymatic activity appeared as clear bands over the dark background.

### 2.10. Cell Cycle Analysis

Flow cytometry analysis of PI-stained cells was performed to demonstrate the effect of AC on cell cycle progression. Briefly, 2 × 10^6^ H441GL cells were cultured in 10 cm dish and treated with 0, 50, 100, 150 *μ*g mL^−1^ concentrations of ACAE, respectively. Cells were harvested after 48 h of treatment, trypsinized, washed and fixed in 70% ethanol for 30 min. After fixation, cells were resuspended in 1 mL propidium iodide (PI) staining buffer (0.1% TritonX-100, 100 mg mL^−1^ RNase A, 500 mg mL^−1^ PI in PBS) at 37°C for 30  min and the proportion of cells in a particular phase of cell cycle was determined by CellQuest software (FACS Calibur, BD Biosciences, USA).

### 2.11. Annexin V-FITC Flow Cytometric Analysis

Annexin V-FITC apoptosis detection kit (BD Biosciences) was used to detect early and late apoptosis. Annexin V has a strong affinity for phosphatidyl serine which is externalized in the membranes of apoptotic cells. In brief, after 48 h of treatment with ACAE (0, 50, 100 and 150 *μ*g mL^−1^), cells were washed in PBS and resuspended in binding buffer (HEPES-NaOH 10 mM pH 7.4, 144 mM NaCl and 25 mM CaCl_2_). Annexin V-FITC (0.2 *μ*g/*μ*L) and PI (0.05 *μ*g/*μ*L) were added and incubated in dark for 20 min. Cells were then subjected to FACS analysis. At least 10 000 events were recorded and represented as dot plots. Data were analyzed by using FCS V3 software.

### 2.12. Tumor Xenografts and In Vitro Molecular Imaging

Tumour xenografts were established by subcutaneous injection of H441GL cells (1 × 10^5^ cells in 100 *μ*L  PBS) into three groups of severe combined immunodeficient, CB17-SCID female mice and each group contained at least four animals. The first group (as the control group) received subcutaneous injection of H441GL cells (1 × 10^5^ cells in 100 *μ*L PBS) without any further treatments. The second and third groups received oral gavages of ACAE 150 *μ*g (6.25 mg kg^−1^ 3 days^−1^) and 500 *μ*g (31.25 mg kg^−1^ 3 days^−1^), respectively, after tumor inoculation. Tumor volume was detected by using an *in vivo* non-invasively optical imaging system (IVIS-200, Xenogen Corp., Alameda, CA, USA). At the end of the experiment, the mice were humanely sacrificed. Animal care and euthanasia were done with the approval of the Institutional Animal Care and Use Committee (IACUC) at Taipei Medical University.

## 3. Results

### 3.1. ACAE Poses Anti-Proliferative Effect on NSCLC Cell Line H441GL Cells

**Figure 1:**
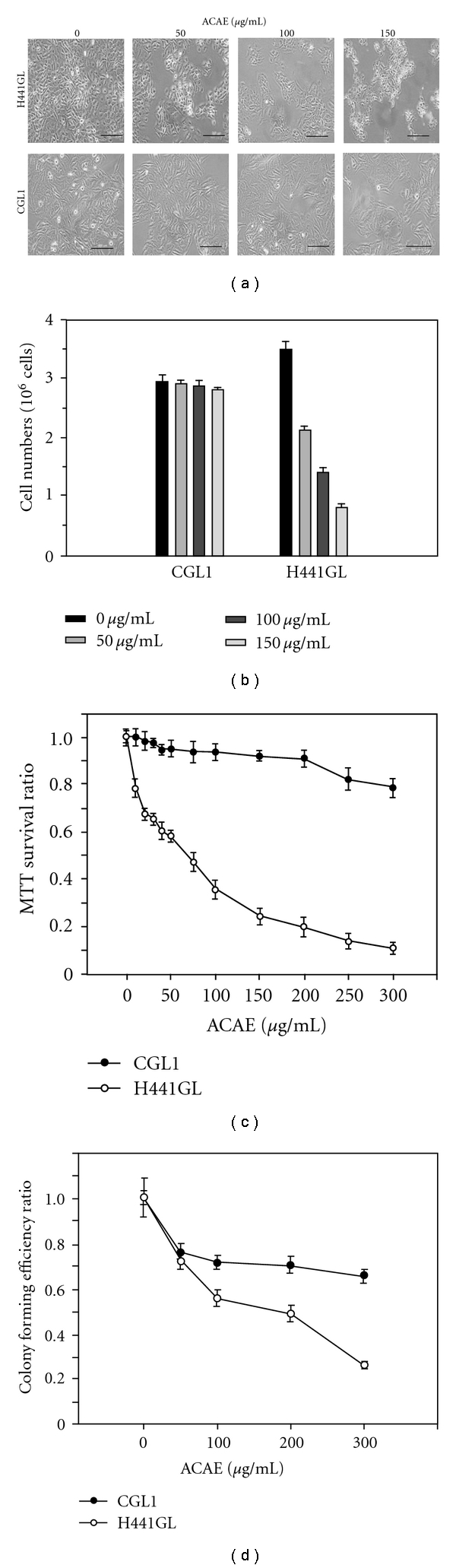
ACAE treatment inhibited the proliferation of H441GL cells. (a) ACAE treatment inhibited H441GL cell growth in a dose-dependent manner but not in CGL1 control cells. ACAE-treated H441GL cells exhibited an abnormal and elongated morphology, while the CGL1 cells remained a normal physiological appearance even at the highest employed concentration of 150 *μ*g mL^−1^. ACAE-mediated anti-proliferative effect was further demonstrated quantitatively by the cell number (b), MTT survival ratio (c) and colony forming efficiency ratio (d) in both H441GL and CGL1 cells. All assays were performed in triplicates and the results were shown as mean ± SD.

To investigate the potential usage of ACAE in the treatment of lung cancer, NSCLC cell line H441GL and control CGL1 cells were treated with different concentrations of ACAE for the examination of ACAE's effect on these cells. In response to ACAE treatment, H441GL cells exhibited an elongated morphology with irregular cytoplasmic volume and decreased cell density (Figure 1(a), upper panel). In contrast, the morphology of CGL1 cells remained unaffected even at the highest concentration of ACAE (Figure 1(a), lower panel), suggesting that ACAE functions on physiological deregulated cells. Next, the proliferation profiles of ACAE-treated H441GL and control CGL1 cells were examined and compared. After a 48 h incubation period, the total number of H441GL cells decreased as the concentration of ACAE increased while CGL1 cells remained relatively unaffected (Figure 1(b)). Similarly, data from the MTT survival and colony forming efficiency, both indices for cell proliferation, exhibited a negative correlation between the MTT survival ratio (Figure 1(c)), colony forming efficiency (Figure 1(d)) and the concentration of ACAE.

### 3.2. Promotion of Cell Cycle Arrest in H441GL Cells by ACAE Treatment

**Figure 2:**
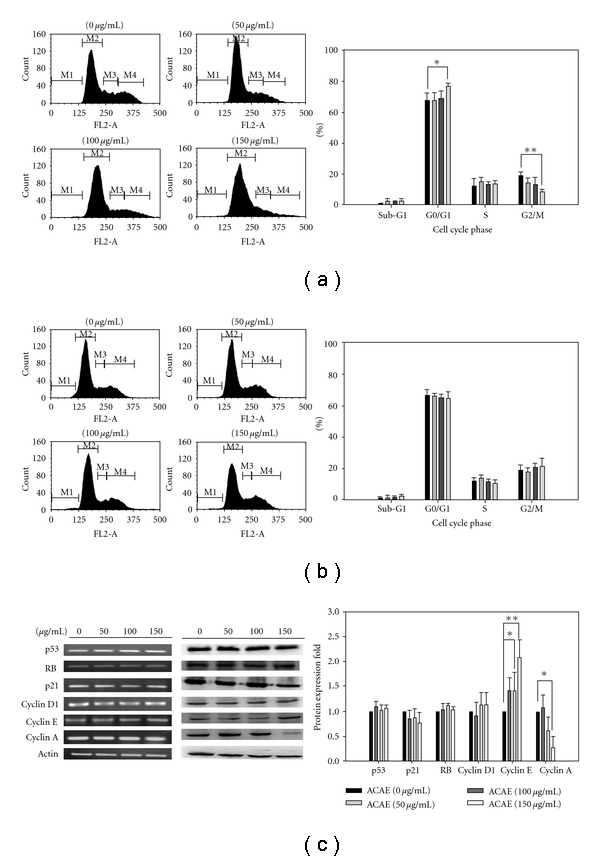
ACAE treatment for 48 h promoted cell cycle arrest in H441GL cells. Representative cell cycle profiles of ACAE treated H441GL (a) and CGL1 (b) cells, respectively, demonstrating arrest in G0/G1 phase. *X*-axis (FL2-A) represents cells stained positive for PI while the *Y*-axis for cell counts. The percentage of cells in each phase is as indicated. (c) Cell cycle regulator molecules at transcriptional ((c), left) and translational ((c), middle) levels were examined. No significant differences were observed at the transcriptional level. However, cyclin A protein expression level decreased while cyclin E increased at 150 *μ*g mL^−1^ of ACAE. Quantitative data representation of western blotting experiments in ((c), right). Each experiment was performed in triplicates (*P < .05, **P < .01).

Next, we examined whether ACAE-mediated anti-proliferative effect on H441GL cells was the result of cell cycle arrest. ACAE-treated H441GL and control CGL1 cells underwent fluorescence-activated cell sorting (FACS) analysis. At 150 *μ*g mL^−1^ of ACAE treatment, the proportion of H441GL cells increased in G0/G1 phase (from 67.02 ± 5.11% to 77.10 ± 1.48%) while decreased in G2/M phase (from 18.84 ± 2.31% to 8.13.10 ± 1.59%), an *∼*10% phase shift when compared to non-treated H441GL cells (Figure 2(a)). In comparison, CGL1 cells appeared to be unaffected by ACAE treatment because the percentage of cells in each stage of division cycle remained approximately unchanged (Figure 2(b)). We also analyzed the expression profiles of several key cell cycle regulatory proteins including p53, RB, p21, cyclins D1, E and A using both semi-quantitative PCR and western blotting techniques. No significant changes in mRNA and protein levels in H441GL cells across the ACAE concentration spectrum examined (Figure 2(c)). However, at the highest concentration ACAE (150 *μ*g mL^−1^), the expression level of cyclin E was found increased while cyclin A decreased (Figure 2(c), left), which corroborated to the increased percentage of cells in G0/G1 phase and decreased proportion of cells in G2/M phase, respectively. Data from western blot analysis were quantitatively represented in fold changes (Figure 2(c), right).

### 3.3. Induction of Apoptosis in ACAE-Treated H441GL Cells

**Figure 3:**
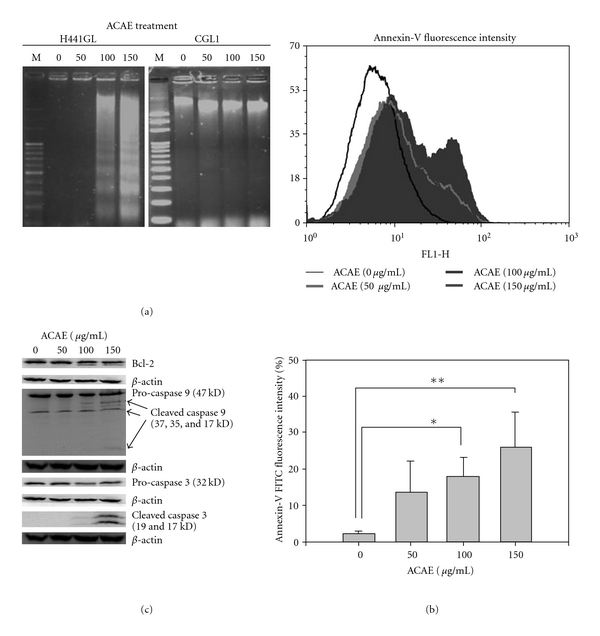
Induction of apoptosis in H441GL cells by ACAE treatment. (a) DNA agarose gel analysis. Genomic DNA samples were obtained from H441GL cells after incubation with different concentrations of ACAE. DNA fragmentation was observed in samples incubated with 100 and 150 *μ*g mL^−1^ of ACAE, whereas DNA samples from CGL1 cells remained intact. Approximately 5 *μ*g of DNA samples were loaded in each lane. (b) Induction of apoptosis was analyzed by flow cytometry with anti-Annexin-V antibody. The intensity of Annexin-V signal in the H441GL cells increased as the concentration of ACAE increased. Quantitatively, the intensity of Annexin-V fluorescence detected in H441GL cells 48 h post-ACAE treatment was concentration dependent (lower panel, *P < .05; **P < .01). (c) Effect of ACAE on apoptotic pathways in H441GL cells. H441GL cells were treated with various concentrations of ACAE for 48 h. A concentration-dependent activation of both caspas-9 and its effector caspase-3 and the down-regulation of Bcl-2 were observed.

After establishing that the addition of ACAE to H441GL cells inhibited their proliferation and caused cell cycle arrest, we then investigated the possibility of ACAE in triggering apoptosis in H441GL cells. After 48 h treatment of various concentrations of ACAE, genomic DNA samples were collected from both H441GL and CGL1 cells. At 100 and 150 *μ*g mL^−1^, ACAE was able to induce DNA fragmentation in H441GL cells but not in CGL1 cells (Figure 3(a)). In supportive of these data, ACAE-treated H441GL cells exhibited a concentration-dependent increase in Annexin-V fluorescence signal when examined by flow cytometric method where an *∼*9-fold increase in Annexin-V signal was detected in H441GL cells treated with 150 *μ*g mL^−1^ of ACAE (Figure 3(b)). Furthermore, we demonstrated a dose-dependent up-regulation of both caspases 9 and 3 in ACAE-treated H441GL cell lysates (Figure 3(c)). Results from these aforementioned experiments indicated ACAE triggered apoptosis in H441GL cells.

### 3.4. Retardation of Migratory Ability of H441GL Cells Prompted by ACAE Treatment

**Figure 4:**
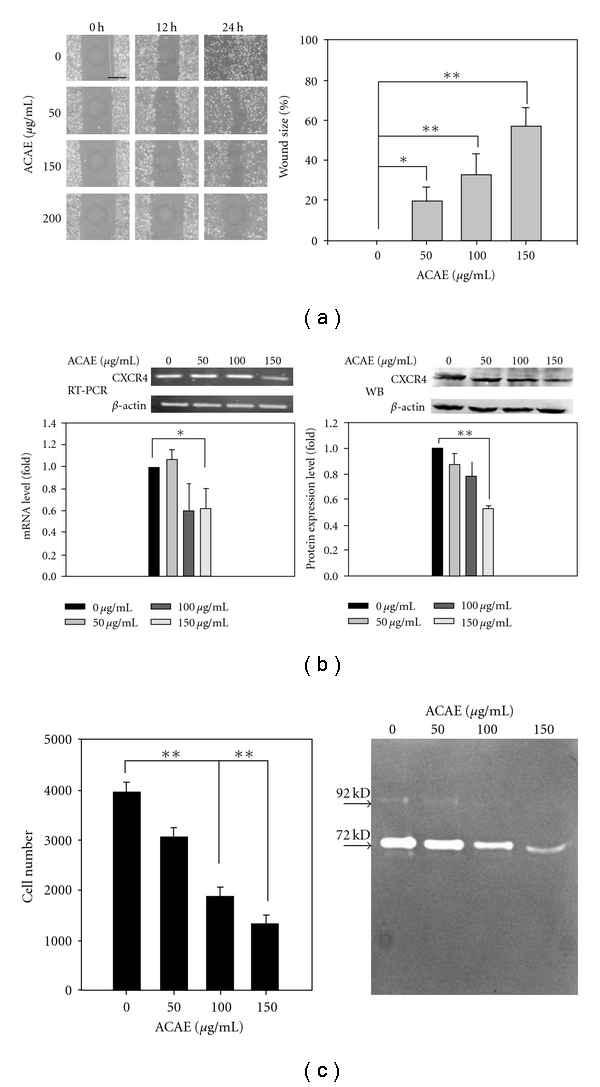
*In vitro* inhibition of migration/invasiveness of H441GL cells by ACAE. (a) Wound-scratch assay data revealed that ACAE exerted a dose-dependent inhibitory effect on H441GL invasive ability after 24 h treatment period. Bar, 500 *μ*m (left). Quantitatively, at 150 *μ*g mL^−1^ of ACAE, a 60% of motility inhibition was observed (right, *P < .05; **P < .01). (b) Semi-quantitative analysis of ACAE-treated H441GL cell lysate demonstrated that CXCR4 transcript level (left) was down-regulated at 150 *μ*g mL^−1^ of ACAE. The lower part represents data expressed quantitatively by densitometric means. Western blot analysis of ACAE-treated H441GL cell lysate probed with anti-CXCR4 antibody (right). CXCR4 expression was down-regulated by ACAE treatment in a concentration-dependent manner. When represented quantitatively (lower), 150 *μ*g mL^−1^ of ACAE exhibited the greatest suppression of CXCR4 expression (**P < .01). (c) Invasive ability of H441GL cells was suppressed by ACAE treatment as demonstrated by transwell analysis (left, **P < .01), while matrix metallo*-*protease (MMP) zymography demonstrated (left) that both pro-MMP9 (92 kD) and pro-MMP2 (72 kD) enzymatic activity in H441GL cells was suppressed by ACAE in a dose-dependent manner.

One of the desirable traits of an anti-cancer agent is the ability to suppress cellular migration. Thus, we set to examine if ACAE could function in modulating migratory ability in H441GL cells. When H441GL cells were treated with various concentrations of ACAE for 12 and 24 h in wound scratch assay, the migratory ability of H441GL cells was increasingly suppressed as the concentration of ACAE elevated (Figure 4(a), left). Overnight treatment of ACAE at 150 *μ*g mL^−1^ resulted in an *∼*60% inhibition on H441GL cells' migratory ability when compared to the control group (Figure 4(a), right). Since CXCR4/SDF-1 chemotactic axis is instrumental in regulating migration in many different cell types, we examined if ACAE-mediated migration suppression was via this axis. Both mRNA and protein expression level of CXCR4 in H441GL cells were reduced by ACAE incubation in a concentration-dependent fashion (Figure 4(b), left and right, resp.). Approximately a 2-fold decrease in CXCR4 expression level was achieved by ACAE at 150 *μ*g mL^−1^ (Figure 4(b), right). The invasiveness of H441GL cells was also suppressed by the addition of ACAE as demonstrated by trans-well analysis (Figure 4(c), left). The decreased invasive ability of H441GL cells was coined with the decreased enzymatic activity of both MMP2 and MMP9 as shown by zymography (Figure 4(c), right).

### 3.5. Non-Invasive In Vivo Monitoring of ACAE-Mediated Anti-tumor Effects on H441GL Tumor-Bearing Mice

**Figure 5:**
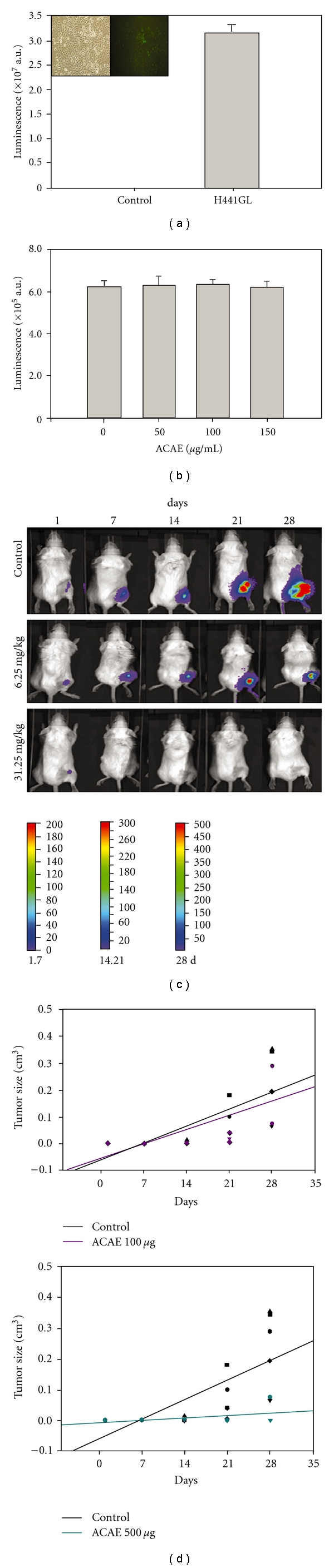
Non-invasive *in vivo* bioluminescence evaluation of ACAE-mediated anti-tumor effect. (a) *In vitro* bioluminescence examination of H441GL cells. H441GL cells demonstrated a substantially higher level of bioluminescence than control H441 cells. The insert depicts H441 control and H441GL cells under fluorescence microscope (GFP used for cell-sorting purpose). (b) ACAE treatment does not interfere with firefly luciferase activity in H441GL cells. (c) Control animals inoculated (s.c) with 10^5^ H441GL cells, receiving vehicle treatment demonstrated a significantly higher tumor burden as assessed from the increasing intensity of the bioluminescent signals over the period of 28 days. Representative bioluminescence images of mice inoculated with 10^5^ H441GL cells and received two different doses of ACAE oral gavages: 6.25 mg kg^−1^ 3 days^−1^ and 31.25 mg kg^−1^ 3 days^−1^, respectively (equivalent to 100 *μ*g and 500 *μ*g, resp.). Both treatment groups demonstrated a significant reduction in tumor burden when compared to the control group, especially in the mice treated with 500 *μ*g of ACAE. (d) Scatter plots of superficial tumor size over time (measured by calliper) in response to two different concentrations of ACAE treatment.

The anti-tumor effect of ACAE was further verified in H441GL-inoculated mice. H441GL cells were genetically modified to contain enhanced green fluorescence protein and firefly luciferase so that *in vivo* bioluminescence imaging was made possible. Initially, the bioluminescence of H441GL was compared to that of parental H441 cells. H441GL cells demonstrated a substantially higher magnitude of bioluminescence when compared to their parental cells (Figure 5(a)). In addition, the possibility that ACAE treatment could interfere with firefly luciferase and its substrate, luciferin was eliminated by *in vitro* bioluminescence assay. Bioluminescent activity of H441GL cells was not altered under different concentrations of ACAE after a brief incubation period (Figure 5(b)). After validation of our bioluminescence system, mice were then inoculated with 10^5^ H441GL cells subcutaneously and received oral gavages of ACAE at 100 *μ*g and 500 *μ*g (equivalent to 6.25 mg kg^−1^ 3 days^−1^ and 31.25 mg kg^−1^ 3 days^−1^, resp.) as treatment. It is clear that ACAE treatment at 100 *μ*g (Figure 5(c), middle row) significantly reduced the tumour burden when compared to that of control group (Figure 5(c), upper row). At a higher concentration, 500 *μ*g, tumor regression was significant to the level where bioluminescent signals from the tumor were undetectable (Figure 5(c)). The tumor size was also measured using a caliper. A similar trend was observed in that the tumor size regressed over the 28-day ACAE treatment regimen (Figure 5(d)).

## 4. Discussion

Different extraction and cultivation methods of AC have been developed and they result in different enrichment of bioactive ingredients. For instance, only methanol extracts from wild and solid-state cultures of AC contained antihypertensive effects in rats but not water extracts [4]. In this study, we aimed to evaluate potential anti-cancer properties of an AC extract obtained by a newly developed sequential extraction method involving ethanol, hence it is given the name ACAE, in a NSCLC mouse model assisted by bioluminescence imaging.

A NSCLC cell line modified to contain dual reporter genes namely green fluorescent protein (G) and firefly luciferase (L), H441GL, was used as both *in vitro* and *in vivo* model. ACAE exhibits several desirable anti-cancer properties. First, ACAE extract was shown to inhibit the proliferation of H441GL in a dose-dependent manner, supported by MTT and colony forming tests. As the concentration of ACAE increased, H441GL cells took on a more elongated shape accompanied by a lower cell density. According to flow cytometric analysis, ACAE treatment promoted a cell cycle phase shift from G2/M to G0/G1 in H441GL cells but not in the control CGL1 cells. Molecularly, the cessation of proliferation could be attributed to the decreased expression of cyclin A since its disruption leads to inhibition of DNA synthesis in mammalian cells [13]. An increased cyclin E (and a slight decrease in p21 although not statistical significantly) expression profile was observed. This interesting observation appeared to be contradictory to the inhibition of proliferation mediated by ACAE because the over-expression of cyclin E and down-regulation of p21 often correlates to poorer prognosis in patients with different cancers [14–16]. On the contrary, a previous study showed that a decreased level of p21 correlated to a survival advantage in individuals with null p53 ovarian cancer [17]. This similar phenomenon might provide a partial explanation in the case of ACAE-treated H441GL cells since p53 is non-functional in H441GL cells. Another interesting observation was that no significant changes in the expression of retinoblastoma protein, RB (a master molecule in cell cycle regulation), in ACAE-treated H441GL cells. The phosphorylation status of RB and its signalling partners including Cdks, p130, p107 as well as E2F transcription factor all play instrumental roles in the regulation of cell cycle in both dividing and non-dividing cells [18, 19]. Therefore, further investigation in the RB-mediated pathways should provide more insights into the molecular mechanisms responsible for ACAE-mediated cell cycle arrest. Nevertheless, non-invasive *in vivo* bioluminescence analysis demonstrated a significant suppression of tumor growth in mice s.c. inoculated with H441GL cells receiving oral treatment of ACAE. At high dosage (31.25 mg kg^−1^ 3 days ^−1^ of ACAE), bioluminescent signals emitted from the tumor were not detectable 28 days post-inoculation, indicating a strong inhibitory effect of ACAE on H441GL cells. In addition, even this high dosage, the animals appeared healthy and active, suggesting its low cytotoxicity.

The second beneficial property that ACAE offers in the treatment of NSCLC is the promotion of apoptosis in H441GL cells. Increased translocation of membrane phospholipid phosphatidylserine (PS) as represented by increased Annexin-V-FITC signals and DNA fragmentation were observed in ACAE-treated H441GL cells, both implicating apoptosis, although the sub-G1 percentage in ACAE-treated H441GL cells was not significantly increased, which was incompatible with the significant DNA ladder shown in Figure 3(a). This discrepancy is remained to be investigated. To further confirm the apoptosis induction, we examined the expression profiles of Bcl-2, pro-caspase-9, -3 and activated forms of caspase-9 and 3. Evidently, Bcl-2 expression was down-regulated in the presence of increasing concentrations of ACAE. This decreased Bcl-2 expression was accompanied by the concomitant decreased levels of pro-caspase-9, -3 and increased respective activated forms. Thus, ACAE elicited mitochondria-mediated apoptosis could contribute to tumor suppression in addition to aforementioned cell cycle arrest.

**Figure 6:**
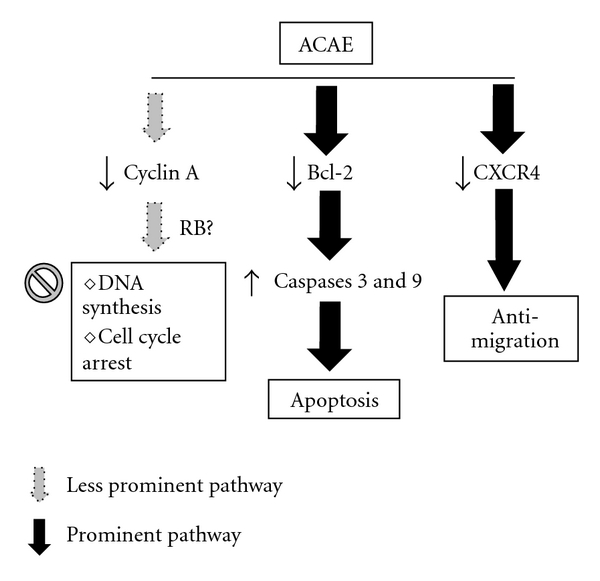
Proposed molecular pathways of ACAE-mediated anti-cancer effects. ACAE-induced anti-cancer effects could be attributed to the combined actions of the cessation of DNA synthesis (cell cycle arrest), apoptosis and anti-migration (later two being more predominant). The protein expression levels of key molecules involved are listed in their respective pathways. Downward and upward arrows depict decreased and increased level of expression.

Finally, another important anti-cancer property of ACAE is that ACAE retards the migratory ability of H441GL cells in a dose-dependent manner as demonstrated by the wound scratch and Trans-well assays. The chemokine receptor CXCR4 and its cognate ligand, stromal cell-derived factor-1alpha (SDF-1), regulate cell trafficking in many different cell types including lymphocyte, mesenchymal stem cell as well as metastatic cancer cells [20, 21]. CXCR4 therefore represents a target for cancer therapy. We observed that ACAE suppressed CXCR4 protein expression in a dose-dependent fashion. It has been shown that in a xenograft mouse model that the down-regulation of CXCR4 leads to the suppression of mammary tumor growth [22]. Another study demonstrated that most pancreatic cancer cells (PaCa) and their stromal fibroblasts express CXCR4 and PaCa-derived CXCL8/IL-8 and fibroblast-derived CXCR12/SDF-1 cooperatively induced angiogenesis *in vitro* by promoting human umbilical vein endothelial cells (HUVECs) proliferation, invasion and tube formation [23]. Furthermore, neutralization of SDF-1 by anti-SDF-1 or anti-CXCR4 monoclonal antibody in pre-clinical studies results in a significant decrease of NSCLC metastases [21]. Therefore, ACAE-mediated down-regulation of CXCR4 expression in H441GL cells could concertedly contribute to the decreased proliferation rate and migratory ability. A proposed model summarizing ACAE-mediated anti-cancer properties is depicted in Figure 6.

Although the exact composition of ACAE is not yet available, it has been reported and suggested that the major anti-inflammatory and anti-cancer component identified are the triterpenoids [3, 24, 25]. Triterpenoids have been shown to inhibit proliferation [24, 25] and induce apoptosis [26–30] in bronchial epithelial cells and NSCLC. Triterpenoids, especially oleanolic acid (OA) and ursolic acid (UA) are found to be anti-inflammatory and anti-tumorigenic *in vivo* [9, 10]. It is important to note that ACAE used in this study contains *∼*12% oleanolic acid by weight (data not shown). We speculate that the relatively high OA concentration in the ACAE contributes to its anti-cancer properties both *in vitro* and *in vivo*. Additionally, a potent synthetic triterpenoid derivative, 2-cyano-3,12-dioxooleana-1,9(11)-dien-28-oic acid (CDDO) has been shown to inhibit the proliferation of various malignant cells independent of p53 status [31]. This is very similar to our observation that ACAE inhibits proliferation in H441GL cells with non-functional p53. The abilities to suppress proliferation and induce apoptosis in H441GL cells by ACAE fit the pleiotropic nature of triterpenoids. Thus, it is plausible that the major anti-cancer attributes of ACAE are derived from its high content of triterpenoids.

Considering the genetic and epigenetic complexities of an advanced carcinoma, combined multifunctional agents/drugs such as ACAE (in this study), triterpenoids, rexinoids, selective estrogen-receptor modulators (SERMS) and deltanoids [32–34] could target multiple signaling pathways in the prevention and treatment of cancer. Bioactive ingredients in ACAE (triterpenoids and others) most likely function through activities including anti-inflammatory, anti-oxidative, immunological and down to transcriptional modulations on the cells to provide anti-cancer effects. All of these overall processes are highly relevant to carcinogenesis and its control via the pleotropic nature of ACAE to ameliorate the multiple defects in the cells and tissues in NSCLC offers a greater possibility of success than the use of a monofunctional agent that targets specifically a single defect.

Collectively, our data indicated that ACAE contains desirable anti-cancer properties. AC has been used extensively as complementary and alternative medicine (CAM) and health supplements in Taiwan, and no reported toxicity in rodents was observed even at a high concentration of 500 mg^−1^ kg^−1^ day^−1^ (unpublished data). Thus, ACAE reported in this study could represent a potential new class of multifunctional CAM for the treatment of NSCLC. Finally, we advocate and endorse the idea of using molecular imaging as a powerful tool for a more comprehensive pre-clinical evaluation of CAM agents.
